# An affordable, quality-assured community-based system for high-resolution entomological surveillance of vector mosquitoes that reflects human malaria infection risk patterns

**DOI:** 10.1186/1475-2875-11-172

**Published:** 2012-05-24

**Authors:** Prosper P Chaki, Yeromin Mlacha, Daniel Msellemu, Athuman Muhili, Alpha D Malishee, Zacharia J Mtema, Samson S Kiware, Ying Zhou, Neil F Lobo, Tanya L Russell, Stefan Dongus, Nicodem J Govella, Gerry F Killeen

**Affiliations:** 1 Ifakara Health Institute, Coordination Office, Kiko Avenue, Mikocheni, PO Box 78373, Dar es Salaam, United Republic of Tanzania; 2 Liverpool School of Tropical Medicine, Vector Group, Pembroke Place, Liverpool L3 5QA, UK; 3 London School of Hygiene and Tropical Medicine, Keppel Street, London WCIE 7HT, UK; 4 Boyd Orr Centre for Population and Ecosystem Health, College of Medicine, Veterinary Medicine and Life Sciences, University of Glasgow, Glasgow G12 8QQ, UK; 5 Department of Mathematics, Statistics, and Computer Science, Marquette University, Milwaukee, WI 53201-1881, USA; 6 Eck Institute for Global Health, University of Notre Dame, Notre Dame, IN 46556, USA; 7 James Cook University, School of Public Health, Tropical Medicine and Rehabilitation Sciences, Cairns, 4870, Australia

## Abstract

**Background:**

More sensitive and scalable entomological surveillance tools are required to monitor low levels of transmission that are increasingly common across the tropics, particularly where vector control has been successful. A large-scale larviciding programme in urban Dar es Salaam, Tanzania is supported by a community-based (CB) system for trapping adult mosquito densities to monitor programme performance.

**Methodology:**

An intensive and extensive CB system for routine, longitudinal, programmatic surveillance of malaria vectors and other mosquitoes using the Ifakara Tent Trap (ITT-C) was developed in Urban Dar es Salaam, Tanzania, and validated by comparison with quality assurance (QA) surveys using either ITT-C or human landing catches (HLC), as well as a cross-sectional survey of malaria parasite prevalence in the same housing compounds.

**Results:**

Community-based ITT-C had much lower sensitivity per person-night of sampling than HLC (Relative Rate (RR) [95% Confidence Interval (CI)] = 0.079 [0.051, 0.121], P < 0.001 for *Anopheles gambiae s.l.* and 0.153 [0.137, 0.171], P < 0.001 for Culicines) but only moderately differed from QA surveys with the same trap (0.536 [0.406,0.617], P = 0.001 and 0.747 [0.677,0.824], P < 0.001, for *An. gambiae* or *Culex* respectively). Despite the poor sensitivity of the ITT per night of sampling, when CB-ITT was compared with QA-HLC, it proved at least comparably sensitive in absolute terms (171 versus 169 primary vectors caught) and cost-effective (153US$ versus 187US$ per *An. gambiae* caught) because it allowed more spatially extensive and temporally intensive sampling (4284 versus 335 trap nights distributed over 615 versus 240 locations with a mean number of samples per year of 143 versus 141). Despite the very low vectors densities (Annual estimate of about 170 *An gambiae s.l* bites per person per year), CB-ITT was the only entomological predictor of parasite infection risk (Odds Ratio [95% CI] = 4.43[3.027,7. 454] per *An. gambiae* or *Anopheles funestus* caught per night, P =0.0373).

**Discussion and conclusion:**

CB trapping approaches could be improved with more sensitive traps, but already offer a practical, safe and affordable system for routine programmatic mosquito surveillance and clusters could be distributed across entire countries by adapting the sample submission and quality assurance procedures accordingly.

## Background

Recent successful malaria control efforts have overwhelmingly relied on proven intra-domicilliary vector control interventions, such as long-lasting insecticidal nets (LLINs) [1-7] and indoor residual spraying (IRS) [8-11], that kill mosquitoes feeding or resting inside houses [12]. Although these indoor interventions have proven potential to reduce *Plasmodium falciparum* transmission and associated disease burden, neither of these alone is sufficient to even approach elimination in endemic areas [13-18] because of persistent vector populations that rest outdoors (exophilic), feed outdoors (exophagic), or feed on animals (zoophagic) [15,18-20]. National Malaria Control Programmes (NMCPs) presently face the challenge of monitoring declining transmission levels mediated by dramatically altered residual vectorial systems with greater sensitivity than ever before. This task will become more challenging as universal coverage with LLINs and IRS is achieved, sustained and even supplemented with additional complementary measures [12,15]. Such residual transmission is often persistent, self-sustaining and quite localized, and may be perennial in some hotspots [21-26], necessitating the implementation of sensitive, longitudinal and extensive vector surveys. Traditional entomologic-monitoring tools have been designed and evaluated for research purposes, primarily in the holoendemic settings where malaria research has traditionally been based. These tools may, therefore, be impractical to apply on scales large enough to detect and target such hotspots of low, but persistent transmission.

Most malaria-endemic developing countries are challenged with a persistent shortage of expertise relating to vector control, and indeed to health systems generally [27-31]. These deficiencies have resulted in weak monitoring, evaluation and management of vector-borne diseases, including malaria. Even if large numbers of expert personnel were available to staff large, predominantly vertical, vector surveillance programmes, the cost of sustaining such human resources would be prohibitive in most African countries [32-34]. Thinking among public health practitioners has therefore shifted to consider devolving the responsibility for vector surveillance and also control to members of the respective communities [32,33,35,36]. This is envisaged to have two advantages: First, this strategy is anticipated to be affordable and can therefore be sustained indefinitely on large scales. Secondly, community involvement is thought to be an effective way for promoting quick uptake and communal support for accountable, politically-viable, public health programmes [32,33,35-40].

Of the numerous options for supplementing LLINs and IRS with complementary vector control measures [12], is the historically-established strategy of larval source management [33,36,40-43]. Larval source management embraces environmental management and the regular application of insecticides to aquatic habitats [44-46] which have not or cannot be modified or eliminated because of their ownership or function [47]. The efficacy and effectiveness of larviciding has recently been evaluated in a range of research and programmatic settings, on scales varying from small rural villages [48-50] all the way through to extensive tracts of a large city [39,51]. The Urban Malaria Control Programme (UMCP) in Dar es Salaam, Tanzania represents an example in which larviciding was implemented on large scales by local government actors through sustainable and affordable systems embedded in routine municipal services [32,39,52]. Specifically, the UMCP implemented three main routine tasks, (1) routine aquatic habitat surveillance, (2) regular application of microbial larvicides and (3) adult mosquito monitoring [39,51]. All these activities are implemented by community owned resource persons (CORPs) assigned to well defined areas of responsibility that the CORP ideally lives in or close to [39,52-54] and that are typically smaller than 1 km^2^[55,56].

**Figure 1:**
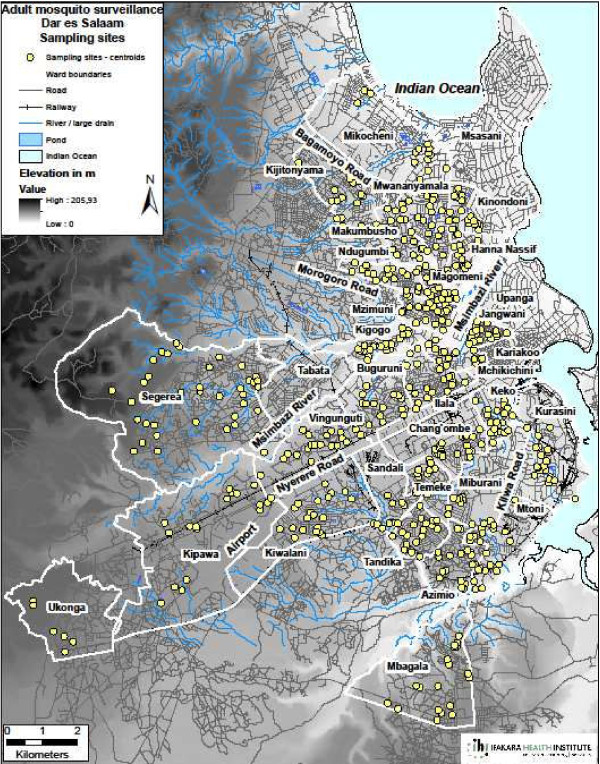
Map of Dar es Salaam showing the wards and respective locations where community-based adult mosquito surveillance was conducted.

While this article focuses on the third activity, namely surveillance of adult mosquitoes, the spatial extensiveness and temporal intensiveness required of this monitoring platform are defined by the challenges of comprehensive larval surveillance and control [57]. Specifically, habitats must be searched for and treated on a weekly bases because microbial larvicides have little residual effect [58] and *Anopheles gambiae* complex mosquitoes develop from egg to adult in less than seven days, in habitats that can be ephemeral and difficult to detect [47,59-61]. It is therefore essential to independently monitor adult vector densities so that gaps in larval surveillance and control [53,54], as well as influx of dispersing vectors from neighbouring areas can be detected. While larval surveillance is clearly required to rapidly respond to such dynamic ecology, such surveys only report on known habitats and locally potential to generate adult mosquitoes. To enable evidence-based, responsive management of the large, decentralized community-based (CB) labour force, which executes larval control on a daily basis [49], an equally spatially- extensive (Figure 1) and temporally-intensive surveillance system is required [39,55,56]. To address this need, the UMCP conducted routine monitoring of adult mosquitoes densities as the primary, most direct indicator of programme performance on a weekly basis [39,51].

The initial monitoring system utilised outdoor human landing catch (HLC) because it was the only method known to reliably catch *Anopheles* malaria vectors with satisfactory sensitivity in this setting [39]. The previous system consisted of a team of 67 CORPs who conducted monthly surveys of 268 locations distributed across 55 km^2^ of Dar es Salaam with a population of >600,000 people [39,51,55,56,62]. Each CORP was assigned four sites in one particular neighbourhood (*mtaa*), one of which was surveyed each week by HLC for one night. Although this interim transmission monitoring system using HLC did produce useful surveillance data, the laborious nature of implementing this community-based scheme on the ground and the vertical management system required to maintain reliable performance were costly and difficult to sustain indefinitely as a routine activity [63]. Moreover, the potential health risks associated with exposure to potentially infectious mosquito bites during human landing catches necessitated the development of a mosquito trapping method which is not only more scalable, affordable and practical [63-65], but also safe for the operator [66].

The Ifakara Tent Trap (ITT) [63-66] was developed to address these specific problems and operates passively all night long without skilled personnel using a single human volunteer who simply sleeps in the tent to act as bait. A number of efficacy studies with the B-model confirm that it is the only reasonably sensitive alternative to HLC [64,65] in urban Dar es Salaam and a small scale pilot study indicated that it is effective in the hands of CB staff with minimal supervision [63]. Furthermore, the latest C-model has been shown to fully protect the user and may even be more sensitive [66].

This paper reports on an evaluation of the effectiveness of a novel extensive and intensive decentralized system for routine entomological surveillance, in which the C design of the ITT was applied by community-based personnel. The effectiveness of this decentralised system was contrasted with an independent quality assured centralized system applying both ITT-C and HLC. The results of these alternative decentralized and centralized surveys were compared with cross-sectional household malaria infection surveys to assess their respective epidemiological relevance in the same set of sampled locations.

## Methods

### Study area

Dar es Salaam is a hot, humid coastal city and experiences two rainy seasons: the short rains from mid-October to early-December followed by the long, more intense rains from March to June. Dar es Salaam is Tanzania’s biggest and most economically important city with an estimated population of 3.3 million in 2010, living within an administrative region of 1,400 km^2^[67,68]. The city is divided into three municipalities, namely Kinondoni, Temeke and Ilala, and these municipalities are further divided into a total of 72 wards. The study site encompasses 31 administrative wards at the heart of the city, comprised of one set of 15 wards previously described as the UMCP study area [51] and another 16 neighbouring wards, totalling approximately 2.65 million residents living in an area of 160 km^2^[67]. Before the initiation of larviciding, the area experienced modest malaria transmission rate with an entomological inoculation rate (EIR) of approximately one infectious bite per person per year [39,51]. The main malaria vectors are members of the *An. gambiae* complex, which prefer to feed outdoors and may therefore be only moderately vulnerable to control with indoor-targeted insecticidal means such as ITNs [62,69].

### The Dar es Salaam UMCP

All UMCP activities are coordinated by the City Medical Office of Health, and fully integrated into the decentralized administrative system of Dar es Salaam [32,39]. The UMCP operates on all six administrative levels of the city: the city council, the three municipal councils it oversees, the 15 wards chosen from those municipalities, containing 67 neighbourhoods referred to as *mitaa* in Kiswahili (singular *mtaa*, meaning literally street), and more than 3000 housing clusters known as ten-cell-units (TCUs), each of which is subdivided into a set of plots corresponding largely to housing compounds [39,51,56]. The main tasks of the three upper levels within UMCP are programme management and supervision, whereas actual mosquito larval surveillance and control is organized at ward level and implemented at the level of TCUs and their constituent plots. In principle, a TCU is a cluster of ten houses with an elected representative known as an *mjumbe*, but typically comprises between 20–100 houses in practice [55]. As a prerequisite for effective management of a larviciding programme, the UMCP implemented routine larval habitat surveillance between 2004 and 2008 [39,53,54]. From March 2006 to date, the UMCP implemented regular larviciding of all mosquito breeding habitats as a means to kill aquatic mosquito stages, prevent adult emergence and reduce malaria incidence and prevalence through a community-based but vertically managed delivery system [32,39,52-54]. UMCP began systematic larviciding in three wards (one from each municipality) in April 2006 [51-54], following complete participatory mapping of the area [55,56] and CB baseline surveys of the breeding habitats. The programme subsequently scaled-up larvicide application to nine wards in May 2007. In March 2008 the programme was extended to all the 15 wards of the original study area. In this particular study, community-based adult mosquito surveys were set up across the original 15 UMCP wards plus an additional 16 adjacent wards from outside the study area to include non-UMCP wards chosen from the same three municipalities where there was no larviciding taking place. Overall, this 160 km^2^ area contained 31 wards, 85 *mitaa,* approximately 8,000 TCUs and approximately 2.65 million residents (Figure 1).

### Routine programmatic adult mosquito surveillance by community-based personnel

Based on a pilot-scale evaluation in 12 wards that used the B-design ITT [63], a CB scheme for trapping adult mosquitoes using the C-design ITT [66] was developed and implemented as a replacement for the previous system that relied on HLC [51]. ITT-C differs from the earlier ITT-B prototype, in that the netting panel lying between the entry funnels and the bait host is bisected into two compartments within the trap. This enables a person in the process of collecting mosquitoes to stand up within the trap while protected from mosquito bites. In addition, there are two long sealable cotton sleeves hanging from each trap chamber to enable operators to safely remove mosquitoes by using mouth aspirators while protected from bites. In contrast, the B design required the opening of the long zipper across the netting panel and aspirating from within the open trap chamber, thereby exposing the operator to mosquito bites [66].

The entomological survey was initially set up across the previous 15 UMCP intervention wards, each of which comprised of a cluster of 20 sampling sites, making a total of 300 sentinel sites distributed across the UMCP study area that were routinely surveyed on monthly basis. This was primarily meant to serve as a tool for routine monitoring of progress of the larviciding programme activities by identifying areas with residual vector populations and, presumably, malaria transmission. Adult mosquito surveillance was therefore decentralized to ward level to coincide with management practice for concurrent community-based larval surveillance and larvicide application. The system adopted a decentralized sampling protocol [63], that enabled unskilled community members, rather than trained entomologists sent from a centralized team, to capture, record and submit mosquito samples, without any night time supervision by the research team, and with only occasional contact with programme staff. This system was modified from that of the original pilot [63] so that only one volunteer per ward was recruited, compared to one per neighbourhood or *mtaa* (3–7 per ward) in the pilot system, to conduct monthly surveys of 20 locations per ward rather than weekly surveys of four locations per neighbourhood (12–28 per ward).

Overall, thirty-one, volunteers including fifteen from the 15 original UMCP wards were recruited and remunerated at a rate of 3500 Tanzanian shillings (2010 US$ 2.70) per night of trapping. Each volunteer took responsibility for trapping mosquitoes for one night per month at each of the 20 locations within his or her assigned ward. They were allowed to choose, at their own discretion, which nights of the week (Monday to Friday) they would sleep in the traps, the sequence they would visit each of their 20 assigned locations, and what time they entered and left the traps, under the condition that they recorded these dates and times in standardized forms. This was considered necessary for promoting a sense of ownership and responsibility for the project, and making working conditions relaxed, conducive and flexible so that the modest remuneration remained sufficiently attractive to retain CORPs and minimize any incentive to fabricate data. Furthermore, there were no consequences to the CORPs for not trapping on a particular night so long as all the 20 sites were sampled at any week day of that particular month. The 20 sampling sites in each ward were deliberately chosen by the local leaders and the CORP, with the intention that they were well-distributed across the ward, close to obvious *Anopheles* larval habitats, and preferably within walled compounds so that safety of the sleeping volunteer was assured.

The volunteers were supplied with all the necessary materials including paper cups, air-tight containers, aspirators, petroleum ether and bicycles for transport. This allowed them to continuously trap, collect and store mosquitoes for a period of one week, recording their observations and trapping sequence daily on a form they were provided with. Samples were submitted each week to the central laboratory for further processing using the bicycles that each CORP was provided with to assist them in moving the trap between the sites within the ward. Each night the trap was erected outside of the designated house and the volunteer slept in it over night to act as a bait to attract human-feeding mosquitoes. Note that the user is completely protected by the fine netting trap chambers where the mosquitoes are trapped [66]. Mosquitoes were removed from the trap chambers using aspirators, transferred into paper cups, and then anesthetized with a small ball of cotton wool soaked in petroleum ether. Dead mosquitoes were then transferred into an air-tight (1.5 ml Eppendorf tubes, Nantong Shenhua Laboratory Apparatus Co., Ltd) container half-filled with silicagel for storage and preservation before submission to the central mosquito laboratory each week. To control for and minimize data fabrication by CORPs, standardized forms were supplied ( Additional file 1: Table S1) and they were obliged to record the approximate number of each relevant mosquito taxon caught, early each morning immediately after they finished collecting, and to document confirmation of his visit with the signature of the house owner where the trapping took place that particular night. At the laboratory, the samples were received by a technician who verified their content before formally recording their acceptance in good condition in a registry book.

This protocol for routine CB sampling with ITT-C across the original 15 UMCP wards, where larviciding had already been established as a routine activity, began in February 2009 whereas the 16 non-intervention wards outside this area started in October 2009. These additional wards were included as a preparatory step for scaling up city-wide vector surveillance and larviciding, as well as to enable subsequent evaluation of the protocol as applied at large scale across the full range of vector densities found in the city. Overall, this CB system for routine surveillance of mosquito biting intensities spanned over 620 designated sentinel sites (clusters of twenty in each of the 31 wards) of which 615 were actually sampled on a monthly basis in practice (Figure 1).

### Randomized quality assurance entomological surveys

To assess the quality of data collected by the decentralized, routine adult mosquito surveys described above, two quality assurance (QA) adult mosquito surveillance teams were recruited, each comprising five catchers earning slightly more than their counterparts in the routine CB system. The first team, earning 4000 TShs (2010: US$ 3.50 per person per night) was responsible for repeating adult mosquito collection using ITT at five locations scheduled one day after the routine CB mosquito surveillance team had applied the same trapping method in these same locations. The sampling framework for the sites involved randomly selecting five sites from the list of locations where the CB collectors had set their traps the previous night. Therefore, this team was responsible for repeating adult mosquito sampling at randomly chosen locations, over four days of the week (Tuesday to Friday), totalling 20 locations sampled for resurvey by the QA team each week. The second team, earning 8,000 Tanzanian Shillings (2010: US$6.15) per day, was responsible for repeating adult mosquito collections using HLC at the same randomly-selected locations used the previous nights for QA-ITT and the night before that for routine CB collections with ITT. This second team worked three days per week (Wednesday to Friday) at the same five randomly chosen locations as the first QA team, totalling 15 locations sampled per week. Outdoor HLC was conducted at each of these houses from 6 pm to 7 am for a period of 45 minutes every hour, allowing for 15 minutes break each hour, as previously described [51,62]. These two QA teams were vertically and regularly supervised, including random night time spot checks by the research team for quality control. The locations selected for QA follow up was not disclosed to either the QA teams nor to the supervising research staff until the day after the routine survey was set up, in the late evening of the day for the first QA surveys using ITT. This was necessary to avoid any possibility of collusion between CORPs in the routine and QA teams and thereby minimize risk of data fabrication. CORPs from the two QA teams were dropped by vehicle at their scheduled stations, accompanied by the field supervisor. The mosquitoes collected by the ITT-C and HLC QA teams were collected by vehicle and taken to the central laboratory the following morning when the catchers had finished their collections.

### Laboratory processing and data reporting

In the laboratory, all mosquitoes were identified morphologically using taxonomic keys [70] as males or females, and as *An. gambiae s.l., Anopheles funestus, Anopheles ziemanni, Culex* species, or *Aedes* species. Abdominal status was scored as gravid/semi-gravid, fed or unfed for all the *Anopheles* and for Culicines. All *Anopheles* caught were subsequently desiccated over silica gel and kept at room temperature until they were further processed. These classification and count data were first recorded on standardized paper forms ( Additional file 1: Table S1) and then reported using mobile phones with specifically designed menus and made available to stakeholders and project staff at the following [71] This web site was also loaded with automatically generated (pre-coded *R* script) weekly synthesis report for the UMCP management staff and other stakeholders to review at will. A wing or a leg of every *An. gambiae s.l*. mosquito caught was analyzed by PCR to identify its exact species within the *An. gambiae* complex [72]. An enzyme-linked immunosorbent assay (ELISA) using a monoclonal antibody that recognizes a repetitive epitope on the circumsporozoite-protein of *P. falciparum* was used to establish malaria sporozoite infection status in each individual *An. gambiae s.l*. specimen [73].

### Cross sectional epidemiological survey

All the 620 sites used for the routine entomological surveillance were mapped to the TCU level [55,56] and the households within each were carefully listed. Three teams of four people, comprised of a supervisor, community-based health nurse and two interviewers conducted the cross-sectional household surveys (March to August 2010) in all households of the house or housing compound (median = 4 households) which routine CB mosquito surveillance was conducted. All people occupying the household were included in the survey, excluding children who were three months old or less. Systematic screening of all the inhabitants of each selected household who were present at the time of the survey, and consented to participate, was carried out to determine their malaria infection status. Parasitological examination was carried out by the community-based health nurses by finger prick with a sterile lancet. A small amount (5 μl) of blood was drawn from consenting residents using micro pipettes and placed on MAL-Pf^®^ (ICT Diagnostics, Cape Town, Southa Africa) malaria rapid diagnostic test kits (RDTs) using histidine rich protein-2 as the test antigen (HRP-2). Such HRP-2 RDTs, including this specific kit, have increasingly been proven sensitive, reliable and accurate for routine malaria diagnosis in the field [74-77]. While this specific test kit is prone to a phenomenon called prozone that results in weak responses to very high density parasitemias, no false negatives were documented in a recent evaluation of this and other comparable HRP-2 based products [78]. Questionnaire responses and RDT results were recorded electronically in the field using Socket SoMo 650 Series (Socket Mobile, Inc) portable digital assistants programmed in Visual CE.

### Data analysis

All the data were entered in coded numeric form, cleaned, restructured and analyzed using SPSS^®^ 18.0 except where described otherwise.

The mean relative sensitivity of the three surveillance methods was estimated by fitting a generalized linear model (GLM) with a negative binomial distribution to the mosquito catch for each recorded trap night, treating surveillance method as a categorical independent variable with location as the subject and date as a within-subject source of variation with first order autocorrelation. Correlation between the mean catch (transformed as logarithm (y + 1)) at each location obtained with the three alternative vector surveillance methods were tested pair-wise using Pearson’s linear correlation test. Associations between the relative sensitivities of CB trapping with ITT and mosquito densities measured by the two QA survey methods were tested for using binary logistic regression [79]. Specifically, GLMs were fitted to the proportion of all mosquitoes caught by the CB-ITT in a given location and week where all methods were applied.

The catches of female *An. gambiae* or *An. funestus* and Culex spp were aggregated by survey method, yielding mean catches for each method per trap night per location. On several occasions, all the three survey methods recoded zero values even after aggregation so an artificial incremental scatter was added to generate the none-zeros and allow separation and visualization of otherwise identical data points. Since divisions by zero gives infinite values, data for each location thus included the sum of several observations of the catches for the specific survey method. In order to establish the density dependence of sampling sensitivity of ITT through either CB or QA methods, the mean catches of the collections by alternative survey methods (CB-ITT and QA-ITT) was divided by the sum of the QA (QA-ITT + QA-HLC) collections, and this denominator was treated as the continuous independent variable in a generalized linear model.

To allow direct comparison of the three surveys in terms of cost-effectiveness only the direct and non-direct expenditures incurred by each system, during the period when all three systems were operating in parallel are considered. These included monthly personnel costs (salaries and volunteer allowances) for each team, supplies and transport costs. Transport costs comprised of the upfront costs for buying a bicycle or a vehicle (for both the CB and QA-surveys, respectively) plus the three years or ten years-depreciated costs (for the bicycles and vehicle, respectively) and their respective monthly-recurrent (service and maintenance) costs. All cost estimates are presented in Tanzanian shillings as recorded at the time they were incurred and then converted into 2010 US$ at a rate of 1408.02 shillings per dollar.

To qualitatively examine differences in age-prevalence profiles associated with malaria transmission hot spots, infection prevalence data from household surveys were initially stratified based on either the presence or absence of any detectable primary vectors (any *An. gambiae s.l.* or *An. funestus* caught) by a given survey method. Subsequently, this approach was refined to stratify on the basis of being amongst the 5% highest mean catches of primary vectors. In all cases, differences between the two strata for each vector surveillance method, in terms of the distribution of infection probability among the following age classes, was tested by *χ*^2^ analysis using Microsoft Excel^®^: less than 5 years, 5 to 19 years and 20 years or more.

Explanatory logistic regression models (GLMM) of malaria infection prevalence were fitted and selected in a forward stepwise manner using *R* version 2.12.2. The association of malaria prevalence with the following independent variables was assessed: mean catch at a given location with each individual entomological survey type, LLIN use, presence of eaves, presence of ceiling, presence of window screening (good indicators of socioeconomic status), larviciding activity, use of insecticide consumer products, travel in the previous month or residence elsewhere, sex and living with both parents. To adjust for spatial and temporal heterogeneities TCU location identity and date were incorporated into all models as random effects. Only variables exhibiting evidence of association with malaria infection risk (P ≤ 0.05) when tested as a single categorical independent variable was retained in the model [80,81]. The variables with the lowest P-value obtained in the exploratory analysis were included first. Based on qualitative examination of age-prevalence relationships in this dataset (see results), this logistic regression analysis was applied only to children and teenagers (<19 years) because the relationship between their exposure and infection prevalence appeared to be higher and to increase with age in areas with higher vector density.

### Ethical consideration and informed consent

The study received ethical clearance from the Medical Research Coordination Committee of the Tanzanian National Institute of Medical Research (Reference numbers NIMR/HQ/R.8a/Vol.IX/279 and 324). Informed consent was obtained from all the participants, including the mosquito catchers and the house owners where the sampling took place, as well as the participants in the household surveys. All the volunteers recruited for conducting HLC were provided with prophylactic treatment with atovaquone-proguanil (Malarone^®^) free-of-charge, which they were obliged to take once a day to prevent malaria infection. In order to deal with the possibility of poor compliance or drug failure, participants in mosquitoes-trapping surveys who developed any symptoms such as fever, chills, headache or nausea, were tested for malaria parasites and would have been offered free treatment if found to be infected but this eventuality never occurred during the study. All participants in either the household surveys found to be infected with malaria were offered supervised treatment with artemether-lumefantrine (Coartem^®^; Novartis Pharma AG, Basel, Switzerland) prescribed by a clinical officer and provided by the community health nurse, following national treatment policies and guidelines, as soon as the RDT test was complete. However, if the participant refused this offer of treatment, they were referred to a nearby health facility and given all required transport and other logistical assistance to attend. Women of child-bearing age found to be infected with malaria were offered treatment with artemether-lumefantrine unless they were known or suspected to be pregnant and in their first trimester, in which case were instead treated with oral quinine as per national guidelines.

## Results

**Figure 2:**
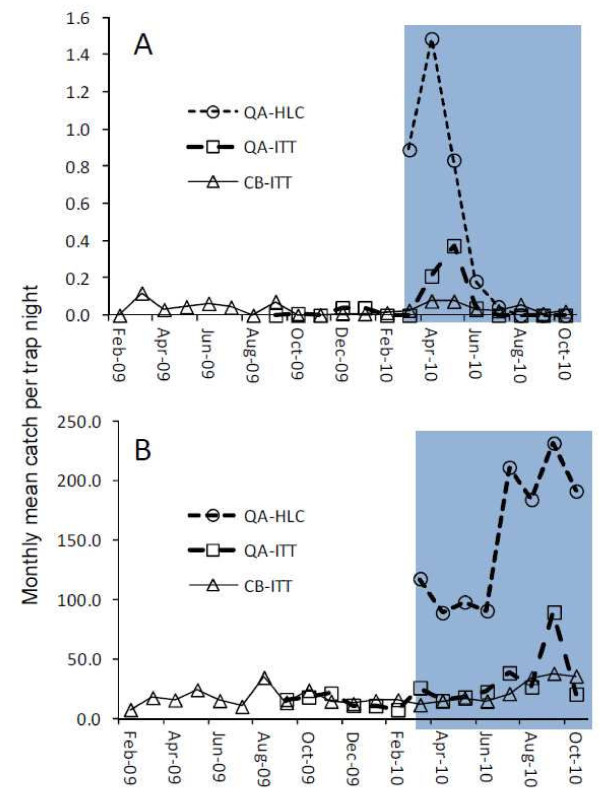
**The monthly mean *Anopheles gambiae *(A) and Culicine (B) densities from the three alternative survey methods being community-based surveys using Ifakara Tent Trap (CB-ITT) and quality assurance surveys based on both human landing catch (QA-HLC) and tent trap (QA-ITT).**

Mean mosquitoes catches by each surveillance system over the course of the study are presented in Figure 2. Of the 372,655 mosquitoes caught by both CB and QA entomological surveillance systems the vast majority (99%) were assorted Culicine taxa: *Culex* spp. (372,161) and *Mansonia* spp. (7). Of the small minority of mosquitoes caught which were *Anopheles* (0.13%; 487), most were *An. gambiae* sl (92.0%; 448) with the remainder comprising *An. funestus* (0.61%; 3) and *An. ziemanni* (7.39%; 36). Consistent with previous reports from this setting [51,63], the majority of *An. gambiae* sl specimens successfully amplified by PCR were *An. gambiae* ss (77.5%; 178) with the remainder being *Anopheles arabiensis* (21.91%; 39). The trapping system had no influence upon sibling species composition (*χ*^2^ = 0.157, d.f. =2, P = 0.924). Both successfully amplified specimens from the *An. funestus* group were *An. funestus* s.s. Only one (0.56%) of the *An. gambiae* ss caught was infected with *P. falciparum* sporozoites.

### Relative sensitivity of alternative survey systems using tent traps

Overall, the sensitivity of ITT-C [66] for trapping both *Anopheles* and Culicines (Table 1) was far lower than HLC when applied by either CB or QA surveys. These relative sensitivity estimates for the C design of the ITT were approximately half of those previously reported for its predecessor, the B design [63-65], for both mosquito taxa. The ITT was less sensitive for both mosquito taxa when applied through the CB surveys than the QA surveys (Table 1) but not dramatically so (Relative rate [95% confidence interval] = 0.536 [0.406,0.617], P = 0.001 for *An.gambiae s.l.* and 0.747 [0.677,0.824], P < 0.001 for *Culex* spp.). However, the mean mosquito catches from the CB-ITT surveys (r^2^ =0.241, P < 0.001**)**, but not those from the QA-ITT surveys (r^2^ =0.012, P = 0.871), positively correlated with those from the QA surveys using the gold standard HLC method.

**Table 1:** Relative sampling sensitivity of community-based (CB) and quality assurance (QA) surveys of mosquitoes with ITT, compared with QA surveys by human landing catch (HLC), as estimated by generalized linear models (GLM)

**Method**	**Number caught**	**Trap nights**	**Locations surveyed**	**Mean trap nights per location**	**Mean Catch [95%CI]**	**Relative rate [95%CI]**	**P**
***Anopheles gambiae s.l.***							
CB-ITT	208	8171	615	13.29	0.026 [0.021,0.033]	0.079 [0.051,0.121]	<0.001
QA-ITT	53	931	293	3.18	0.057 [0.039,0.085]	0.182 [0.101,0.328]	<0.001
QA-HLC	187	335	240	1.39	0.560 [0.385, 0.815]	1.00*	NA
***Culex spp***							
CB-ITT	287,398	8171	615	13.29	20.7 [19.3, 22.0]	0.153 [0.137, 0. 171]	<0.001
QA-ITT	35,642	931	293	3.18	27.1 [23.9, 30.8]	0.215 [0.190, 0. 243]	<0.001
QA-HLC	49,121	335	240	1.39	147.7 [133. 8,163.0]	1.00*	NA

NA: not applicable.

CI: confidence interval.

^*^ Reference category.

**Figure 3:**
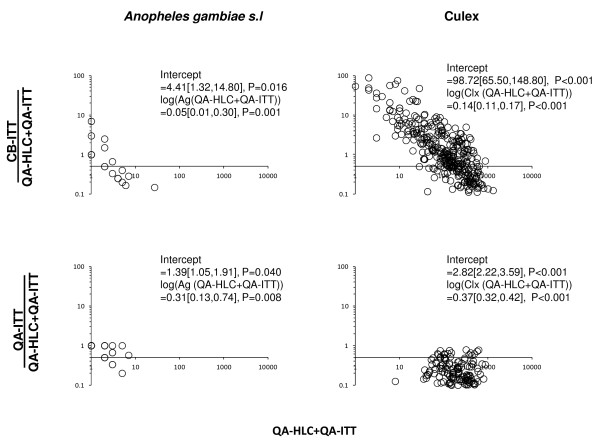
**Density-dependence of alternative ITT-based survey methods relative to the HLC-based QA surveys for sampling *Anopheles gambiae s.l. *(A and C) and *Culex *spp. (B and D).** The density-dependence is illustrated by plotting the catches from alternative methods divided by the corresponding sum of catches from QA-ITT and QA-HLC or both against the absolute CB-ITT catches.

Both the CB and QA surveys with ITT exhibited high density-dependent sensitivity when compared to the gold standard QA surveys with HLC (Figure 3), which is consistent with previous observations [64]. All ITT surveys were clearly less sensitive at high mosquito densities compared to the reference QA surveys with HLC but at very low densities the ITT is at least sensitive than the gold standard HLC. It is notable that not only is the intercept of the plot for the CB-ITT surveys lower than for QA-ITT surveys, the downward slope as mosquito density increases is much steeper (Figure 3). This suggests that high mosquito densities reduce the sensitivity of the ITT, and that standards of practice for its use by CB staff are also adversely affected by high mosquito densities or associated environmental variables, the most obvious of which is rainfall.

**Figure 4:**
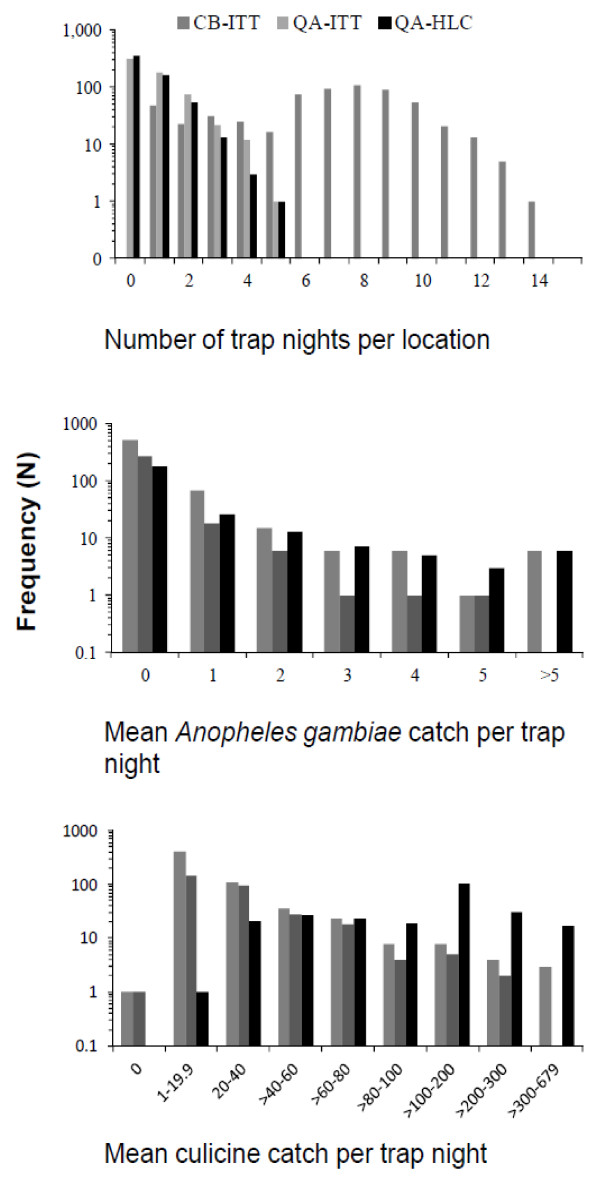
The frequency distributions of the person trap nights and mosquito densities across a range of survey locations by the three surveillance systems.

Despite the much lower average sensitivity of CB surveys with ITT per person night of sampling (Table 1), and declining sensitivity observed as mosquito densities increase (Figure 3), overall CB surveys had slightly greater absolute sensitivity in terms of the total number of mosquitoes caught (Table 2). This occurs because it was possible to maintain these CB surveys in a slightly larger number of locations but, more importantly, because they enabled consistent longitudinal monthly monitoring of mosquito density, resulting in a far greater number of samples per survey location (Figure 4, Table 2). By comparison, the well-controlled QA surveys were clearly more sensitive per person-night of trapping (Table 1) but could only visit any given sites within this large, widely distributed set of locations (Figure 1) on one or two occasions per year (Figure 4).

**Table 2:** **Crude estimates of the costs for each surveillance method per night of trapping and per *An. gambiae s.l *. caught over the selected period outlined in Figure **2** when all three surveillance systems were simultaneous in operation**

**Estimated Parameter**	**Units**	**Community-based**	**Quality assured**	
		**CB-ITT**	**QA-ITT**	**QA-HLC**
Number of samples	Person-nights	4284	457	335
Number caught	No. of *An. gambiae s.l*	171	42	169
Mean catch	No. of *An. gambiae s.l * per person-night	0.04	0.09	0.50
Volunteer costs	TSh	14,994,000	1,828,000	2,680,000
Salary costs	TSh	10,589,820	13,793,820	24,413,820
Transport costs	TSh	3,100,000	20,340,000	20,340,000
Total Expenditure	TSh	28,683,820	35,961,820	47,433,820
Cost per sample	TSh per night of sampling	6,695.57	78,691.07	141,593.49
Costs per specimen of *An. gambiae s.l*.	TSh per *An. gambiae s.l*	167,741.64	856,233.81	280,673.49

All costs are presented in Tanzanian Shillings (TShs). The corresponding estimates of the expeditures in US dollars can be computed at a mean 2010 exchange rate of 1408.02 TShs per US$.

The intensive and extensive sampling frame of the CB surveys was possible because it was the cheapest of the three surveillance systems, costing approximately US$6 per night of sampling, compared to US$72 for running the QA-ITT-C and US$100 for the QA-HLC. In this low transmission setting with very sparse vector populations, entomological transmission surveillance proved an expensive undertaking but CB surveys proved the most affordable approach overall, despite their low sensitivity per person-night of sampling (Table 1). An average of US$163 was spent per specimen of *An gambiae s.l.* caught by the CB surveys, as compared to approximately US$787 and US$199 for QA surveys using ITT and HLC, respectively (Table 2).

### Relationship between mean mosquito densities and malaria infection prevalence

**Figure 5:**
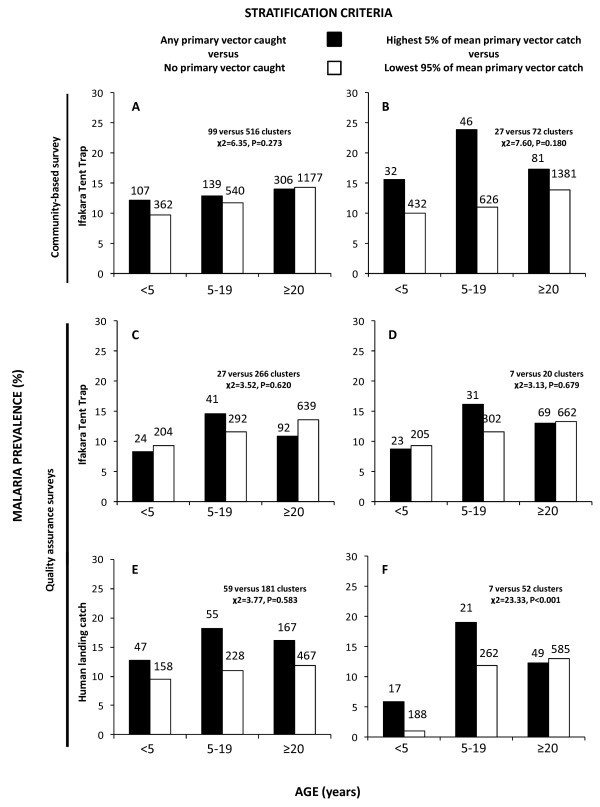
**Age-specific malaria parasite prevalence stratified by mean vector density (*An. gambiae *and *An. funestus *combined) for each mosquito surveillance systems.** For the left hand column (A, C, E), *An. gambiae*-mean catch is stratified as 0 or >0 and for the right hand column *An. gambiae*-mean catch is stratified using the upper and lower ranges being ≥ 0.25, versus ≤ 0.22 for CB-ITT (B), ≥4.00 versus ≤ 3.00 for QA-ITT (D) and ≥1.00 versus ≤0.50 for QA-HLC (F). The number at the top of each bar represents the total number individuals within particular age group from a set stratified surveyed clusters tested for malaria with RDT.

Consistent with the range of vector densities observed in this urban setting (Figure 4), parasite prevalence data from the cross-sectional survey conducted at 357 of the locations confirmed that there was generally moderate transmission across the study area (Figure 5) with an overall prevalence of 13.3% (421/3173). Malaria infection prevalence consistently increased with age (OR [95%CI] = 1.23[1.059,1.392], P = 0.0166), rather than peaking among young children as was observed previously in 2004–06 [51] indicating a loss of age- and exposure-associated immunity, presumably as a result of lowered mean transmission intensity across the area since that time or a reflection of asymptomatic adult infections that usually go unreported but were seen in this survey [82].

When the surveyed locations were stratified by vector density, using the three different survey systems and two alternative stratification criteria, prevalence peaked amongst older children and teenagers in the upper stratum for five out of six of the stratification criteria, and in one case the age-prevalence profile differed significantly between the strata (Figure 5). Further analysis with logistic regression, which allowed us to control for cluster effects associated with the sampled household clusters and the times they were surveyed, was therefore restricted to data from children and teenagers, amongst whom prevalence appears to be consistently positively related to both age and exposure to transmission.

Logistic regression analysis of infection status among residents under twenty years of age revealed that, other than location (P ≤ 0.001) and the time of the survey (P < 0.001), only the mean *An. gambiae* catch obtained from the CB surveys was significant as a predictor of malaria risk (Table 3). The fitted model includes a significant positive intercept for the dependent variable (Table 3). Malaria infection risk was therefore significant even where no primary vectors could be detected (Table 3), suggesting that appreciable malaria transmission amongst residents of Dar es Salaam occurs away from their homes. Baseline infection risk increases with *An. gambiae s.l.* density and a four-fold increase in risk is estimated for individuals living in areas where an average of one *An. gambiae* is caught per person-night of CB surveillance with ITT (Table 3). Neither of the QA surveys of vector density using either ITT or HLC surveys had any appreciable predictive value of malaria prevalence (Table 3). Possible confounders that were tested and then excluded from all the final model included the type of floor, walls and roof (good indicators of socioeconomic status), use of insecticide consumer products, travel in the previous month or residence elsewhere, sex and living with both parents. Interestingly, having both closed eaves and a ceiling (P = 0.532), or having one of them (P = 0.804), or having one of these plus screened windows (P = 0.850) had no apparent impact on malaria risk despite their high levels of uptake arising from the perception that they protect against mosquito bites [51,83]. Using an untreated net (P = 0.607) also had no impact and it is also notable that neither of the interventions previously shown to confer protection [51], namely use of an LLIN (P = 0.094) or living in an area covered with larviciding (P = 0.428) had any significant protective effect or improved the model fit. Similarly, none of the three observed house characteristics, namely type of floor (P = 0.5432), wall (P = 0.7602) and roof (P = 0.3694), as well as the use of personal protection measures, such as insecticide consumer products including mosquito coils (P = 0.3839), topical repellents (P = 0.2566), or insecticide sprays (P = 0.2799) had significant effect nor impact on the goodness of fit of model.

**Table 3:** ***Anopheles gambiae *mean catch per night as risk indicator for malaria parasite prevalence among children and teenagers (<20 years of age) as determined by fitting separate logistic regression models (GLMM) to data from each of the three survey methods**

**Survey type**	**OR [95%CI]**	**P**
*Community-based with ITT* mean *An. gambiae s.l. * catch	4.43 [1.091,17.956]	0.0373
Intercept	0.096[0.075,0.123]	<0.0001
*Quality assurance* with ITT mean *An. gambiae s.l. * catch	1.01[0.465, 2.178]	0.989
Intercept	0.102[0.076,0.136]	<0.0001
*Quality assurance* with *HLC *mean *An. gambiae s.l.* catch	0.94[0.823, 1.081]	0.448
Intercept	0.111[0.080,0.151]	<0.0001

See Table 2 for details of sample sizes for each entomological survey data set. Note that for all three models location and date included in the models were also highly significant random effects.

## Discussion

Community-based use of the ITT with no supervision from the research team proved the most cost-effective and epidemiologically relevant way to monitor adult malaria vector mosquitoes and was also safer than the HLC gold standard method. Although this approach has low relative sensitivity per night of sampling, it is also by far the least expensive and allows far more intensive longitudinal sampling so that it is slightly more effective than even QA-HLC in terms of absolute sensitivity, cost-effectiveness and spatial extensiveness. Critically, the ability to conduct longitudinal sampling on a monthly temporal cycle that is sufficiently frequent to capture seasonal variation in vector density at hundreds of locations concurrently gives this implementation system epidemiological predictive value that traditional survey methods, relying on closely supervised research teams, did not even distantly approach (Table 3).

This CB survey achieved a spatial resolution of one trap-night sample per 0.27 km^2^ every month and 0.93 km^2^ every week across the 31 volunteers and their assigned wards. In demographic terms, this is equivalent to one trap night for every 5,848 residents per month or 21,739 residents per week. Such intensive and extensive monitoring of adult mosquito responds to the needs of the local UMCP larviciding programme because it is matched to the scales to which responsibility for applying larvicides is devolved so that gaps in coverage, sensitivity and quality of these activities can be identified and rectified. The distribution of adult mosquito sampling locations therefore encompassed the assigned target areas of every person responsible for larvicide application so that their individual personal performance can be evaluated objectively and independently, based on one or more observations each month. In spite of the proven efficacy of larvicides [84,85], the success of a larviciding programme relies on the sensitivity of detection and treatment of all potential larval habitats by large numbers of widely-distributed staff managed in a decentralized way at ward level [53,86]. This spatially extensive, community-based surveillance with the ITT has demonstrated the potential for identifying malaria transmission hotspots on very fine scales (Table 3). Longitudinal CB surveillance with the ITT or any other practical, ideally more sensitive, alternative trapping technology may be a useful means for mapping residual vector populations and enable targeted control with supplementary vector control measures such as larval source management that complement LLINs or IRS. An ideal trap is presumably low cost, less bulk, easily transportable and preferably independent of electrical power.

Although various traps and survey platforms have been developed and implemented for trapping, monitoring and studying mosquito vectors of malaria and other disease in various parts of the world [87-93], currently declining malaria transmission levels [4-6,94,95] and mosquito densities [17] pose a particular challenge to monitoring and evaluating disease trends. To date, mosquito vector surveillance has mostly depended on the use of conventional trapping methods applied under strict research-controlled settings, with very few reports of application through community-based platforms. Research-controlled studies are often limited in scope in terms of spatial and temporal coverage due to associated high running costs and therefore very expensive to maintain on scales large enough to detect hot spots of persistent transmission levels occurring on very fine scales and support decisive management of vector control activities. This is exacerbated by the limited number of expert personnel in most malaria endemic countries. Even when community based surveys have been implemented with conventional tools, the quality of unsupervised data collection has been a concern to many public experts. In this study, the ITT was used to sample mosquitoes at a much higher spatial resolution as an outdoor trap. In comparison with other recently reported surveys using window exit traps (Table 4), the use of ITT appears to be more user-friendly and affordable because it is less disruptive and intrusive to householders since it is set up outside of the house. While all the survey platforms described in (Table 4) successfully engaged local communities in their operations, only this approach developed in Tanzania includes external quality assurance mechanisms.

**Table 4:** Comparison of the surveillance system described in this paper with some published large scale and longitudinal entomological surveys using window exit traps (WET), Ifakara tent traps (ITT) and human landing catches for monitoring malaria vector populations

**Study and location**	**Surveillance tool**	**Implementation platforms**	**Quality assurance**	**Number of cluster**	**Sampling sites per cluster**	**Trap-nights per month**	**Temporal scale (Trap nights)**	**Duration of the surveys**	**Total number of trap months**
**Abilio et al. 2010** Zambezia province, central northern Mozambique	WET	Community-based (home owner) as stand alone	No	19	6	114	788	2006-2007 and 2009-2010	48
**Sharp et al. 2007** Bioko Island, Equatorial Guinea	WET	Community-based (home owner) as stand alone	No	16	6	96	59,307	2004-2005	24
**Chaki et al.** (Urban Dar es Salaam, Tanzania)	ITT and HLC	Community-based (community volunteers)	Yes	31	20	615	8,171	Feb 2009- Oct 2010	20

All survey systems compared here were based on monthly sampling intervals.

Despite the advantages that the tent trap and community-based survey system appear to offer, both the ITT technology and the delivery system described here have significant limitations, some of which synergize negatively. The ITT has important limitations as an entomological and epidemiological surveillance tool because of limited sensitivity, particularly at high mosquito densities (Figure 3 and reference [64]). The observation that this problem is exacerbated when used through the CB system presumably reflects our informal observations of the poor compliance by the CORPs with setting up and sleeping in the traps during wet season peaks of mosquito density when rain may enter the trap. Moreover, the bulky nature of the trap makes it impractical for indoor use and therefore unsuitable for surveying the proportion of human exposure to mosquito bites that occurs indoors. Even for outdoor applications, the space requirements of the trap poses particular challenges in densely populated informal settlements in urban settings. Moreover, even with the predominantly flat topography of Dar es Salaam, the bulkiness of the trap makes it too heavy and difficult to be moved between sampling locations by one volunteer without at least a bicycle.

## Conclusions

As the global malaria elimination initiative [94,96-100] advances, spatially extensive longitudinal vector surveillance systems, such as the CB trapping system reported here, will become increasingly necessary to characterize sparse residual vector populations across large areas, and for monitoring and evaluating impact of interventions upon them. In practical terms, we recommend that further advances with CB mosquito surveillance systems will require development of improved trap technologies that will ideally no longer require human bait. Such products should be more sensitive, less bulky, less expensive, and should readily trap the outdoor-biting, zoophagic mosquito species that increasingly dominate residual transmission across the tropics [18-20,101]. Several experimental prototypes already exist that use synthetic odour mixtures as bait and are highly efficacious for sampling a broad spectrum of mosquito species [102-106], including some that representatively samples the taxa that attack humans [107]. This study, therefore recommends that such evaluated trap designs can be adapted for the surveillance of a variety of mosquito-borne diseases including malaria, lymphatic filariasis and dengue fever.

## Competing interests

We declare that none of the investigators has any conflict of interest. None of the funders had any role in the evaluation design, data collection, analysis, interpretation, drafting of the manuscript or decision to publish.

## Authors’ contributions

PPC took the lead in designing and implementing the study, data analysis and in writing of the manuscript. YM, AM and DM participated in the implementation of various aspects of the adult mosquito and household parasitology surveys as well as data management. NJG, ADM, ZJM, TLR and SK supported the design and implementation of the various study aspects including data management. GFK was involved in designing the larviciding system that these vector monitoring systems are intended to support. YZ and NFL supported the PDA programming, data collection and management. SD contributed substantially to producing the maps and drafting of the manuscript. GFK supervised all aspects of the study design, implementation, data analysis and drafting of the manuscript. All authors read and approved the final manuscript.

## Supplementary Material

Additional file 1 ** Table S1.** Standardized UMCP forms for routine adult mosquito surveillance teams to help control for and minimize data fabrication by CORPs. Click here for file
