# Supplementary material for: An affordable, quality-assured community-based system for high-resolution entomological surveillance of vector mosquitoes that reflects human malaria infection risk patterns
Source: Malar J. 2012 May 24;11:172. doi: 10.1186/1475-2875-11-172 (PMC3475008; doi:10.1186/1475-2875-11-172)
Supplement: Additional file 1 — Table S1. Standardized UMCP forms for routine adult mosquito surveillance teams to help control for and minimize data fabrication by CORPs. [file 1475-2875-11-172-S1.docx]

Standardized UMCP forms for routine adult mosquito surveillance teams to help control for and minimize data fabrication by CORPs

| URBAN MALARIA CONTROL PROGRAM | | | | | | | | | | | |
| --- | --- | --- | --- | --- | --- | --- | --- | --- | --- | --- | --- |
| ROUTINE ADULT MOSQUITO SURVEILLANCE WITH IFAKARA TENT TRAP (ITT) | | | | | | | | | | | |
| MUNICIPAL (*Manispaa*) | |  | | | | | | | | | |
| Ward (*Kata*) | |  | | | | | | | | | |
| Name of the volunteer (CORP) | |  | | | | | | | | | |
|  | | | | | | | | | | | |
| Date (tarehe) | *Mtaa* | Ten-cell Unit (shina) | House number (Namba ya Nyumba) | Time of entering the tent (muda wa kuingia hemani) | Time of leaving the tent (Muda wa kutoka hemani) | Number of mosquitoes caught (Idadi ya Mbu aliokamata) | Total number of Anopheles (Idadi jumla ya Anophelesi) | Total number of Culex (Idadi jumla ya Culex) | Total number of Aedes (Idadi jumla ya Aedes) | House owner ‘s name (Jina la mwenye nyumba) | Signature of the house owner (sahihi ya mwenye nyumba) |
|  |  |  |  |  |  |  |  |  |  |  |  |
|  |  |  |  |  |  |  |  |  |  |  |  |
|  |  |  |  |  |  |  |  |  |  |  |  |
|  |  |  |  |  |  |  |  |  |  |  |  |
|  |  |  |  |  |  |  |  |  |  |  |  |
|  |  |  |  |  |  |  |  |  |  |  |  |
|  |  |  |  |  |  |  |  |  |  |  |  |
|  |  |  |  |  |  |  |  |  |  |  |  |
|  |  |  |  |  |  |  |  |  |  |  |  |
|  |  |  |  |  |  |  |  |  |  |  |  |
|  |  |  |  |  |  |  |  |  |  |  |  |
